# Lenticulostriate vasculopathy in newborns: whole genome sequencing data analysis

**DOI:** 10.3389/fped.2025.1531086

**Published:** 2025-08-14

**Authors:** Svetlana Dauengauer-Kirlienė, Dovydas Pranauskas, Yogen Singh, Vaidutis Kučinskas, Alina Urnikytė

**Affiliations:** ^1^ Department of Human and Medical Genetics, Faculty of Medicine, Institute of Biomedical Sciences, Vilnius University, Vilnius, Lithuania; ^2^ Department of Pediatrics, Division of Neonatology, University of California—UC Davis Children’s Hospital, Sacramento, CA, United States; ^3^ Populational Genomics Laboratory, Faculty of Medicine, Translational Health Research Institute, Vilnius University, Vilnius, Lithuania

**Keywords:** lenticulostriate vasculopathy, ultrasound scans, newborns, whole genome sequencing, population

## Abstract

**Objectives:**

Lenticulostriate vasculopathy (LSV) refers to hyperechogenic vessels detected in thalami and basal ganglia, using cranial ultrasound. Awareness of LSV has revealed its links to various neonatal diseases that can affect brain development ante- or postnatally. Congenital infections and hypoxic- ischemic conditions are the main risk factors of LSV. However, precise etiology of LSV remains unknown. The aim of this study was to analyze the whole genome sequencing (WGS) data of newborns diagnosed with LSV to evaluate genetic linkages with LSV manifestation.

**Study design:**

We analyzed whole genome sequencing variation data of newborns with LSV (*n* = 6) and control group newborns (*n* = 19). WGS variation data was annotated using ANNOVAR in GRCh37 (hg19), RefSeqGene, gnomAD, SIFT, dbSNP151, CADD and gerp++gt2. Bash language was used to develop a program that counts variant frequency and compares them between groups.

**Results:**

We identified one exonic nonsynonymous variant putatively associated with LSV, located in *WNK1* gene [NM_213655.5: c.2219T > C p.(Leu740Pro)]. This variant is associated with pseudohypoaldosteronism type 2C and hereditary sensory and autonomic neuropathy type 2A. Pseudohypoaldosteronism can increase blood pressure, resulting in damaged or stiff blood vessels, similar to LSV. The variant is currently classified as a variant of uncertain significance due to insufficient evidence to determine its definitive role in these conditions.

**Conclusions:**

The identification of this unique variant in *WNK1* provides a potential genetic link to the etiopathogenesis of LSV, offering new insights into this condition. However, further functional studies and more comprehensive genetic research are required to establish definitive associations.

## Introduction

Every practitioner performing neonatal head ultrasounds encounters linear, branched echogenic changes in the basal ganglia area, prompting the question: “Is this a normal finding, or is this pathognomonic odd some etiology?” Even for the experienced clinicians, deciding whether to explore this finding thoroughly or dismiss it as an insignificant incidental finding poses a persistent dilemma. Historically overlooked, these subtle positive signs on ultrasonography periodically come to the forefront of neonatal interdisciplinary research (Table 1).

**Table 1:** Studies on lenticulostriate vasculopathy and neurologic outcomes.

Study	Study design	Study population	LSV cohort	GA, weeks	MRI/CT imaging	Conclusions
Wang et al. (1)	Prospective	Infants diagnosed with LSV including cryptogenic and symptomatic groups	34/34	NA	NA	LSV in infancy may predict neuropsychiatric disorders; more severe in symptomatic cases.
Shin et al. (2)	Retrospective	Neonates with varying degrees of LSV severity	32/110	NA	NA	Severe LSV is associated with neurodevelopmental delay.
Maayan-Metzger et al. (3)	Retrospective	Preterm infants with isolated LSV	84/84	25–34	NA	Long-term impact unclear; further investigation needed.
Francovich et al. (4)	Retrospective	Infants with LSV evaluated for developmental outcomes	46/173	NA	NA	Potential increased risk of abnormal development associated with LSV.
Robinson et al. (5)	Systematic Review	Multiple studies involving preterm infants with LSV	–	<32	Applied in some studies	Inconclusive evidence on neurodevelopmental outcomes; mixed results.
Fabre et al. (6)	Retrospective	Infants with clinically severe LSV and additional health monitoring requirements	58/58	25–42	Applied	Severe LSV requires comprehensive follow-up; significant health issues noted.
Sisman et al. (7)	Prospective	Preterm infants diagnosed with LSV in NICU	70/407	≤32	NA	No significant neurodevelopmental differences at 18–36 months corrected age between LSV and control groups.
Chamnanvanakij et al. (8)	Retrospective	Very preterm infants diagnosed with LSV	10/20	<32	NA	LSV associated with poorer cognitive and behavioral performance.
Robinson et al. (9)	Retrospective	Very preterm infants diagnosed with LSV	26/225	<32	NA	No significant differences in neurodevelopmental outcomes at 24 months.

GA, gestational age; LSV, lenticulostriate vasculopathy; MRI/CT, magnetic resonance imaging/computed tomography; NA, not available or not applied; NICU, neonatal intensive care unit.

Lenticulostriate vasculopathy (LSV) is defined radiologically by the presence of echogenic vessels in the striatum, typically visualized by cranial ultrasound during the first year of life (10). Despite absence of uniform agreement on the ultrasonographic evaluation, classification of LSV, and ongoing debates regarding its clinical relevance, these linear echogenicities merit scientific attention and further research to study their etiology and clinical significance. The histological features of LSV typically include endothelial proliferation, fibrosis of vessel walls, and sometimes, microcalcifications, which contribute to the characteristic echogenic appearance seen on ultrasound scans (11). The incidence of LSV is increasing over the years, which varies widely from 0.4% to 32% worldwide (12), which is concerning, especially when its significance remains unknown.

Research studies are suggestive of two possible pathogenetic mechanisms for LSV. Primary LSV involves intrinsic genetic factors, notably linked to significant genomic rearrangements that often lead to serious clinical outcomes, which are highlighted by the clear links between LSV and genetic aberrations (13). Consequently, LSV typically presents as an incidental radiological finding, generally without specific diagnostic significance. However, in certain cases, calcifications in the basal ganglia can become a pathognomonic feature of a syndrome, indicating a more direct clinical relevance (14).

Most of the research has been focused on the secondary pathogenetic mechanism, indicating that environmental factors play a crucial role in the development of LSV. Although there are no direct associations between pregnancy-linked maternal comorbidities and LSV, the relationship with maternal exposure to steroids, antibiotics, and magnesium sulfate continues to fuel controversy regarding the placental pathogenetic role (15, 16). Manifesting both ante- and post-natally, LSV has been linked to infectious and hypoxic insults (15).

The anatomical localization of LSV in the striatum, a critical gray matter structure, underscores the potential for associated neurodevelopmental disorders, neurosensorial hearing loss, and motor dysfunction. Moreover, the unique anatomy of the arteries supplying the striatum heightens their susceptibility to hypoxia and ischemia, leading to further complications (17).

Most studies on LSV include mixed neonatal groups in clinical or hospital settings where health conditions vary. There is a notable lack of specific studies focusing on the incidence of LSV exclusively in a healthy newborn population, as routine head ultrasounds are not typically performed for healthy newborn screening. Despite extensive discussions about risk factors and the role of LSV in individual health prognosis, a significant gap remains in the scientific data regarding its etiopathogenesis. This highlights the need for more research and define a control study population.

To our knowledge, no studies have yet evaluated the genotype-phenotype correlations of LSV within a healthy population. However, the healthy newborns genomes could serve as a reference for the control studies. Since newborns have been exposed to no external factors other than the in-utero environment, they provide an ideal baseline control cohort. Our study aims to assess potential genetic causes of LSV in a healthy newborn cohort using whole genome sequencing data.

## Materials and methods

### Demographic and selection criteria

This prospective case-control study of 25 healthy newborns was conducted as part of the “ANELGEMIA” project (18). Detailed descriptions of the study group population, inclusion criteria, DNA extraction, and whole genome sequencing data processing are available in our previous publication (18). Notably, the inclusion of newborns required the absence of any diagnoses from ICD Chapters “P” (Perinatal Conditions) and “Q” (Congenital Malformations). Consequently, each newborn underwent a comprehensive evaluation post-birth, which included ultrasound (US) screening.

**Figure 1:**
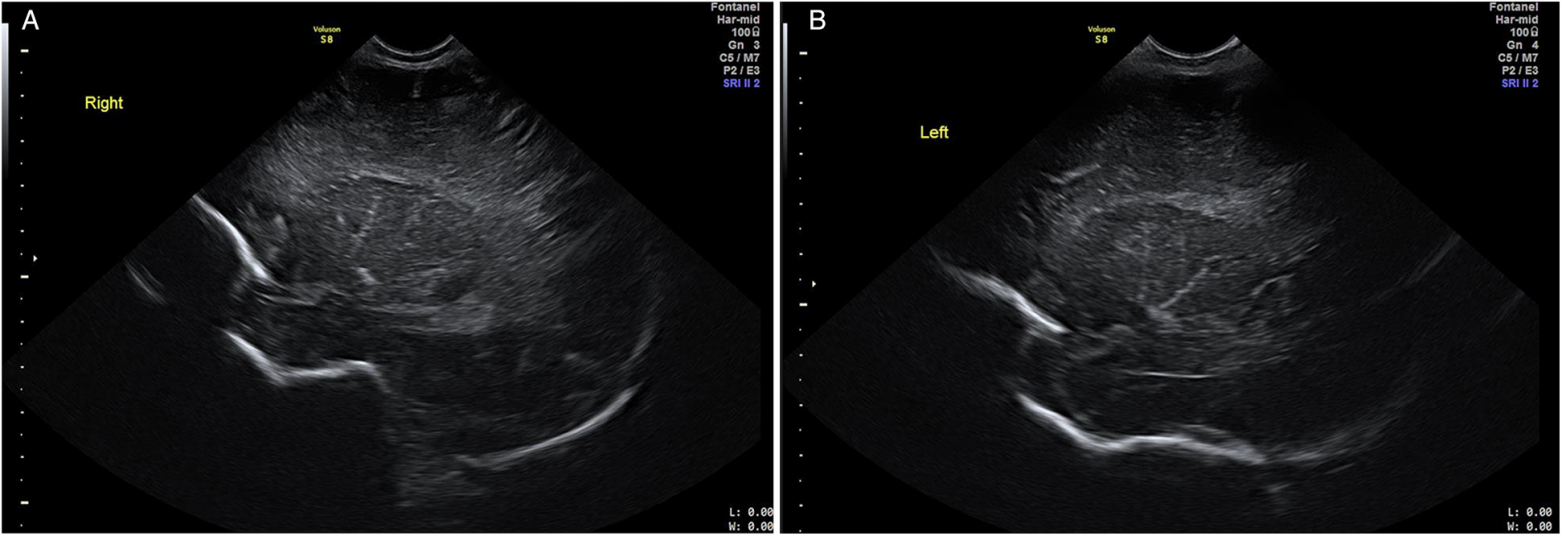
Cranial US images. Right parasagittal (image **A**) and left parasagittal (image **B**) views showing bilateral LSV with at least one hyperechoic MCA branch.

**Figure 2:**
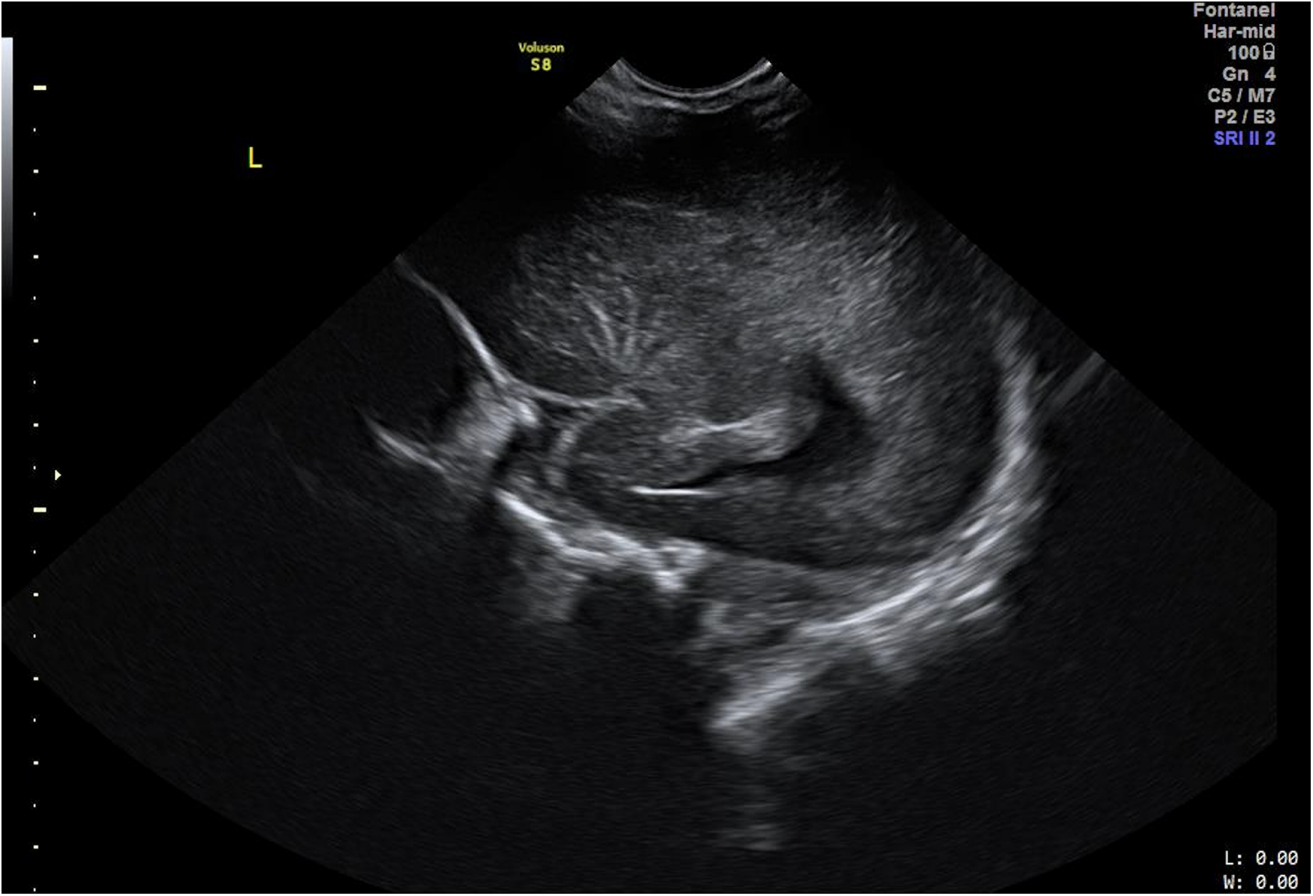
Cranial US image. Left parasagittal view showing unilateral LSV with multiple hyperechoic branches of MCA.

The US evaluations were performed using a GE Voluson S8 machine (GE Healthcare, Chicago, IL, USA) with an 8C-RS probe for initial assessments and a 12l-RS probe for more in-depth evaluations of the brain, operated at 8 Hz and 12 Hz respectively by the clinician experienced in neonatal sonography. All US examinations were performed at 48 h after birth prior to discharge from the hospital. LSV was diagnosed when at least one echogenic branch of the middle cerebral artery (MCA) was observed bilaterally (Figures 1A,B), or when multiple hyperechoic branches were seen in a unilateral parasagittal view (Figure 2). Neonates diagnosed with LSV were assigned to the case group (*n* = 6), while those without significant head US findings were placed in the control group (*n* = 19).

DNA was extracted from whole blood (10 ml) samples using a QIAGEN GENTRA® Puregene® Blood Kit (Qiagen GmbH) according to the manufacturer's protocol. DNA concentration and quality were assessed using a NanoDropR ND-1000 spectrophotometer (NanoDrop Technologies Inc., Wilmington, DE, USA).

WGS and quality control analysis was completed by CeGaT company, located in Tubingen, Germany. 100 ng of genomic DNA was sequenced paired–end using Illumina NovaSeq™ 6,000 Sequencing System at coverage of 26.88–61.38× (an average of 36.27×). Analysis of sequenced genomes was performed using the Illumina DRAGEN platform (v3.6.4). Reads were mapped to the reference human genome hg19. The quality of the FASTQ files was assured using FastQC software.

### Data analysis

Descriptive statistics analyses of case and control groups were performed using R software with the Rcmdr package. Continuous variables, including newborn gestational age, birth weight, mothers’ age, number of pregnancies, and parity, were summarized as median and interquartile range (IQR) due to non-normal distributions. Comparisons between the LSV positive and LSV negative groups for these variables were conducted using the Mann–Whitney *U*-test. Categorical variables, such as sex, mode of delivery and maternal risk factors were presented as proportions (%), and given the small sample size these were compared using Fisher's Exact Test. A significance level of *p* < 0.05 was applied to assess statistical differences.

Bash language was used to develop a program that counts variant frequency and compared them between groups. Firstly, we merged six VCF files, that were generated from newborns with LSV, into a single file (dataset A) and calculated the frequency of genomic variants in the case group (healthy newborns with LSV). Then, we created a file (dataset B) containing all the genomic variants found in the control group by merging 19 VCF files. This file serves as a dataset of genomic variants found in healthy Lithuanian newborns that did not have LSV. Two generated files which contained genomic variants (A and B) were compared. The program identified genomic variants exclusive to file A and marked them as output. They were selected for further analysis.

Gene enrichment analysis was performed using Enrichr (19). The entry list contained genes in which exclusive genetic variants occurred (program's output). Gene duplicates were removed from the entry list. The exclusive case group variants were analysed manually. ClinVar (20) and OMIM (21) databases were used to determine the clinical relevance and pathogenicity of the variants. Variant frequency and in silico scores were also considered.

Segregation analysis was performed for all six families iwhere WGS data were available for both parents and the LSV positive neonate. Genotypes of the candidate *WNK1*:c.2219T > C (p.Leu740Pro) variant were extracted for each trio. Inheritance patterns were evaluated based on Mendelian expectations. A variant was considered Mendelian if the child's heterozygous status could be explained by at least one heterozygous or homozygous carrier parent. Cases where one parental genotype was unavailable, but the other parent carried the variant were classified as “probably Mendelian”. No additional validation (e.g., Sanger sequencing) was performed.

AnnotationWGS variation data was annotated with ANNOVAR v.20210123 (22) using GRCh37 (hg19), RefSeqGene, gnomAD (23) SIFT (24), dbSNP151 (25), CADD v.1.3 (26) gerp++gt2 (27), MutationTaster (28), DANN (29), PhastCon (30), and PhyloP (31).

## Results

Overall incidence of LSV among ANALGEMIA study group was 17,4% (6 of 35). A total of 25 newborns were included in the study, with LSV negative (*n* = 19) and LSV positive (*n* = 6). Comparisons of maternal and newborn characteristics between the groups are shown in Table 2.

**Table 2:** Comparison of maternal and neonatal characteristics between LSV negative and LSV positive groups.

Variable	LSV negative (*n* = 19)	LSV positive (*n* = 6)	*p*-value
Gestational age (weeks)	40 (39–40)	40 (40–40)	0.7593
Birth weight (grams)	3,680 (3,480–3,815)	3,510 (3,350–3,730)	0.5036
Sex (*n*, %)	13 Boys (68.4%)	3 Boys (50%)	0.63
Natural vaginal delivery (*n*, %)	19 (100%)	5 (83.3%)	0.24
Mothers’ age (years)	32 (30.5–34.5)	27.5 (27–28.75)	0.003963
Number of Pregnancies	2 (1–3)	1 (1–1.75)	0.0848
Parity	2 (1–2)	1 (1–1.75)	0.3727
Gestational diabetes (*n*, %)	5 (26.3%)	2 (33.3%)	1
Hypothyroidism (*n*, %)	2 (10.5%)	2 (33.3%)	0.234

This table summarizes the characteristics of the LSV negative and LSV positive groups. Data are presented as median (IQR) for non-normally distributed variables and proportions (%) for categorical variables; statistical comparisons were performed using Mann–Whitney *U*-test for continuous variables (gestational age, birth weight, mothers’ age, number of pregnancies, and parity) and Fisher's Exact Test for categorical variables (sex, mode of delivery, gestational diabetes, and hypothyroidism) due to their non-normal distributions and the small sample size; *p*-values indicate the statistical significance of differences between the two groups, with a *p*-value <0.05 considered statistically significant.

There were no statistically significant differences in most characteristics between the groups. Both groups had similar gestational ages (*p* = 0.7593) and birth weights (*p* = 0.5036). The distribution of sex (*p* = 0.63) and natural vaginal delivery (*p* = 0.24) were also comparable across the groups. While mothers in the LSV positive group were younger (median = 27.5 years, IQR 27–28.75) compared to the LSV negative group (median = 32 years, IQR 30.5–34.5, *p* = 0.003963), there were no significant differences in number of pregnancies (*p* = 0.0848), parity (*p* = 0.3727), or maternal risk factors such as gestational diabetes (*p* = 1) and hypothyroidism (*p* = 0.234). Overall, the lack of significant differences in most maternal and newborn characteristics suggests that the two groups are comparable, allowing for a reliable evaluation of genetic variables in the context of LSV.

By merging cases' group VCF files and selecting variants that are present in all of the groups' newborns, we created a dataset with 1,650,351 genetic variants. After comparison between case and control group datasets, 3,469 genomic variants were exclusive to the case group and were used for further analysis. Enrichment analysis showed a connection between genes in which genomic variants occurred and aldosterone synthesis and secretion. With this information in mind, genomic variants were filtered using OMIM and ClinVar databases. In ClinVar database, variants that were classified as benign or likely benign were removed. In OMIM dataset, genomic changes that were associated with blood pressure changes, calcium homeostasis or small brain vessel diseases were selected as candidate variants. We identified one exonic nonsynonymous variant putatively associated with LSV, located in *WNK1* gene [NM_213655.5: c.2219T > C p. (Leu740Pro)]. This variant is associated with pseudohypoaldosteronism type 2C (PHA2C) (OMIM #614492) and hereditary sensory and autonomic neuropathy type 2A (HSAN2A) (OMIM #201300). Importantly, this genomic variant is rarely found worldwide. The highest frequency of this variant in the European population is 0.00001134 (13/1146712), specifically in the non-Finnish European population. Single instances of the variant have also been detected in East Asian (1/40,888) and African American/African populations (2/72,882) and this genomic variant has not yet been found in other populations (32). While the variant resides in a gene with plausible biological links to vascular and developmental pathways, its pathogenicity remains inconclusive without further functional validation. Table 3 represents the computational and database annotations for the *WNK1*:c.2219T > C (p.Leu740Pro) missense variant identified in the LSV-positive cohort. The variant affects a highly conserved genomic region and is exceedingly rare, with no homozygotes reported and a global allele frequency of 0.002% in gnomAD. It meets the ACMG PM2 criterion (moderate evidence for pathogenicity based on rarity). The *in silico* prediction results are mixed. Some tools predict a benign effect, suggesting limited impact on protein structure or function. Others indicate a potentially deleterious role, highlighting the possibility of biological relevance. Conservation-based tools emphasize that the altered region is evolutionarily constrained, which may imply functional importance. Splice prediction analysis does not suggest any effect on mRNA processing.

**Table 3:** Summary of genetic variant associated with LSV.

Variant	Gene	Phenotype	Rs ID	Prevalence	"In silico” scores	Clinvar
NM_213655.5: c.2219T > C p.(Leu740Pro)	WNK1	PHA2C/HSAN2A	rs72649847	0.0000192	SIFT—0.22 (T)	VUS
					MutationTaster—1.0 (D)	
					DANN—0.987 (D)	
					CADD—11.89	
					GERP—5.39	
					PhastCons—1.0	
					PhyloP30—1.138	
					REVEL—0.06 (B)	
					MetaLR—0.07 (B)	
					BayesDel—−0.11 (B)	
					SpliceAI—0 (No splice effect)	

HSAN2A, hereditary sensory and autonomic neuropathy type; 2A PHA2C, pseudohypoaldosteronism type 2C; B, benign; D, deleterious; T, tolerated; VUS, variant of uncertain significance.

In addition, inheritance analysis of the *WNK1*:c.2219T > C (p.Leu740Pro) variant revealed a consistent Mendelian pattern across all informative families (Table 4). In six LSV-positive trios, the variant was present in a heterozygous state (0/1) in all affected neonates. Parental genotypes confirmed that in five of six families, the variant was inherited from one heterozygous or homozygous carrier parent. In one case, the paternal genotype was not available, however, maternal heterozygosity supports probable Mendelian transmission. No cases of *de novo* occurrence were identified, further suggesting that this variant is not a sporadic finding but rather inherited in a pattern consistent with autosomal transmission.

**Table 4:** Segregation analysis of the *WNK1*:c.2219T > C (p.Leu740Pro) variant in LSV-positive trios.

	Variant	Chr	Pos	rsID	Ref	AlT	Mother	Father	Sibling	Inheritance
Family no	T > TC	12	974,355	rs72649847	T	TC				
002							1/1	0/1	0/1	Mendelian
005							1/1	0/1	0/1	Mendelian
014							0/1	0/1	0/1	Mendelian
021							0/1	N/A	0/1	Probably Mendelian
025							0/1	1/1	0/1	Mendelian
032							0/1	1/1	0/1	Mendelian

## Discussion

The etiology and pathogenesis of LSV remain largely unexplored. Most previous studies have focused on the epidemiology, distribution among different infant populations, and the clinical prognosis of LSV (Table 1). To date, no comprehensive genetic studies have been conducted to investigate the underlying genetic causes of this condition. Existing sparse literature on the genetic aspects of LSV is primarily composed of small case reports involving dysmorphic or severely affected patients, where LSV is more often noted as a co-occurring finding rather than the main subject of interest (33, 34). Moreover, the metabolic patterns associated with LSV have rarely been the focus of genetic research or discussions (35).

Based on existing literature that links LSV to fluctuations in blood pressure, we hypothesized that genomic rearrangements may contribute to the appearance of these small yet visible vascular anomalies. Our study identified a genomic variant in the *WNK1* gene, which encodes protein kinase WNK1. The WNK signaling pathway plays a critical role in regulating blood pressure through its effects on renal function and vascular tone (36). *WNK1* is a key regulator in the WNK kinase cascade, influencing processes such as cell volume, cell growth, blood pressure regulation, and neural signal transmission (37). Variants in this gene have been implicated in disrupting ion transporters and channels responsible for maintaining sodium and potassium balance. This disruption indirectly affects aldosterone action, leading to alterations in blood pressure regulation (38). Moreover, mutations in *WNK1* can lead to dysregulated ion transport in vascular smooth muscle cells, which may cause abnormal vasoconstriction or vasodilation, contributing to hypertension (36). Studies in mouse models show that *WNK1* knockout results in embryonic lethality, emphasizing its essential role in embryogenesis, particularly in angiogenesis (39). Furthermore, WNK1 dysfunction may impair endothelial function, increasing the risk of vascular complications such as arterial stiffness and a reduced ability to dilate in response to blood flow (40). These pathophysiologic mechanisms in mouse models support our findings and shows potential LSV phenotype-genotype relationship. Additionally, the identified variant is linked to monogenic disorders such as PHA2C and HSAN2A (25). PHA2C is characterized by excessive sodium reabsorption and reduced potassium excretion, leading to hypertension and hyperkalemia despite normal aldosterone levels (41, 42). On the other hand, HSAN2A is a rare autosomal recessive genetic disorder often leading to severe injuries or limb amputations due to progressive sensory loss (43). Interestingly, both these conditions manifest in early childhood or adolescence.

While *WNK1* has known roles in vascular biology and embryogenesis, as evidenced by mouse models where *WNK1* knockout results in embryonic lethality and disrupted angiogenesis (39), the specific *WNK1* variant identified in this study (c.2219T > C, p.Leu740Pro) does not currently meet established criteria for pathogenicity. The observation of Mendelian inheritance across all informative families suggests that this variant is inherited rather than a *de novo* finding. This pattern, while not indicative of causality, supports the idea that such variants may act as modifying factors or component of broader polygenic or context-dependent mechanism in LSV pathogenesis. It also emphasizes the importance of considering inherited variants—even those with uncertain clinical significance—in exploratory analyses of subtle neonatal phenotypes. Further functional studies in this field would be helpful. A key strength of our study lies in its novelty and comprehensive evaluation. This is the first study comparing the findings with a case-control cohort. Routine head US screening is not generally recommended for healthy newborns, yet it revealed the presence of LSV in 17.1% of term-born neonates, a frequency similar to that reported in studies involving sick infants. This underscores the need for further clinical and epidemiological studies to better understand the prevalence and implications of LSV in otherwise healthy populations. Moreover, despite the relatively small size of our cohort, the study benefits from its homogeneity, with no significant differences in descriptive statistics or known risk factors that might influence the development of LSV. This uniformity provided an opportunity to identify and emphasize the relevance of a rare genomic rearrangement that may play a role in LSV pathogenesis. Finally, this study represents the first genome-wide analysis conducted in a healthy population, aimed at identifying potential genetic pathways involved in the development of LSV.

We acknowledge several limitations of our study. First, we focused on the genomic variants that appeared six times in the case group and not at all in the control group. This approach may have overlooked variants that occurred less frequently in the control group but were still potentially significant. Moreover, this approach assumes only a single genomic variant as the possible biological pathway, while it is likely that multiple variants could influence its development. Secondly, the small number of LSV-positive cases (*n* = 6) significantly limits the statistical strength of variant discovery and validation. Additionally, the global rarity of the identified WNK1 variant further reduces the likelihood of drawing firm conclusions about genotype-phenotype links. Finally, the lack of long-term follow-up data on the participants limits our ability to draw conclusions about the future health outcomes of these infants with LSV. Taken together, these limitations highlight the need for larger studies with more comprehensive control for potential confounders to assess the relevance and reproducibility of the genetic findings reported here.

## Conclusion

Lenticulostriate vasculopathy is often detected as part of a broader diagnosis, making it challenging to interpret and understand its clinical significance. In this study, a variant in the *WNK1* gene was identified through phenotype-based analysis in six infants diagnosed with LSV with no other associated comorbidities or abnormalities. Although we do not propose this variant as causal, we consider it a plausible signal worthy of exploration within the biological context of *WNK1*'s role in vascular development and blood pressure regulation. Its exclusive presence in otherwise healthy LSV cases, coupled with its mechanistic relevance, supports its inclusion as a hypothesis-generating finding that may inform future studies. Focusing on genomic variant analysis in larger cohorts of individuals diagnosed with LSV could provide more definitive insights into the contribution of this variant to the development of LSV. Further studies are needed to understand the long-term outcomes in infants with LSV.Understanding the genetic mechanisms behind LSV could help clinicians better assess the clinical significance of LSV, guiding decisions about further care and management. This study provides a foundation for future research into the genetic causes of LSV and similar conditions with unclear etiology, and it highlights the potential of genetic testing in elucidating disease mechanisms, particularly in cases where it is difficult to gather a representative sample.

## Data Availability

The datasets analysed during the current study are available in the Figshare repository, https://doi.org/10.6084/m9.figshare.22952774.
